# Association between knee alignment, osteoarthritis disease severity, and subchondral trabecular bone microarchitecture in patients with knee osteoarthritis: a cross-sectional study

**DOI:** 10.1186/s13075-020-02274-0

**Published:** 2020-09-04

**Authors:** Xuequan Han, Junqi Cui, Kai Xie, Xu Jiang, Zihao He, Jingke Du, Linyang Chu, Xinhua Qu, Songtao Ai, Qi Sun, Liao Wang, Haishan Wu, Weituo Zhang, Zhifeng Yu, Mengning Yan

**Affiliations:** 1 Shanghai Key Laboratory of Orthopaedic Implants, Department of Orthopaedic Surgery, Shanghai Ninth People’s Hospital, Shanghai Jiao Tong University School of Medicine, Shanghai, China; 2 Department of Pathology, Shanghai Ninth People’s Hospital, Shanghai Jiao Tong University School of Medicine, Shanghai, Shanghai China; 3 Department of Bone and Joint Surgery, Renji Hospital, Shanghai Jiao Tong University School of Medicine, Shanghai, China; 4 Department of Radiology, Shanghai Ninth People’s Hospital, Shanghai Jiao Tong University School of Medicine, Shanghai, China; 5 Clinical Research Center, Shanghai Jiao Tong University School of Medicine, Shanghai, China

**Keywords:** Knee osteoarthritis, Hip-knee-ankle angle, Subchondral trabecular bone, Microarchitecture, Micro-CT

## Abstract

**Background:**

Knee osteoarthritis (OA) is a common disabling disease involving the entire joint tissue, and its onset and progression are affected by many factors. However, the current number of studies investigating the relationship between subchondral trabecular bone (STB), knee alignment, and OA severity is limited. We aimed to investigate the variation in tibial plateau STB microarchitecture in end-stage knee OA patients and their association with knee alignment (hip-knee-ankle, HKA, angle) and OA severity.

**Methods:**

Seventy-one knee OA patients scheduled for total knee arthroplasty (TKA) underwent preoperative radiography to measure the HKA angle and Kellgren-Lawrence grade. Tibial plateaus collected from TKA were scanned using micro-computed tomography to analyze the STB microarchitecture. Histological sections were used to assess cartilage degeneration (OARSI score). Correlations between the HKA angle, OA severity (OARSI score, Kellgren-Lawrence grade), and STB microarchitecture were evaluated. Differences in STB microstructural parameters between varus and valgus alignment groups based on the HKA angle were examined.

**Results:**

The HKA angle was significantly correlated with all STB microarchitecture parameters (*p* < 0.01). The HKA angle was more correlated with the medial-to-lateral ratios of the microarchitecture parameters than with the medial or lateral tibia plateaus. The HKA angle and all STB microarchitecture parameters are significantly correlated with both the OARSI score and Kellgren-Lawrence grade (*p* < 0.01).

**Conclusions:**

The STB microarchitecture is associated with the HKA angle and OA severity. With the increase of the knee alignment deviation and OA severity, the STB of the affected side tibial plateau increased in bone volume, trabecular number, and trabecular thickness and decreased in trabecular separation.

## Introduction

Knee osteoarthritis (OA) is an important public health problem and one of the world’s leading disabling diseases [1, 2]. OA is currently considered a whole joint disease involving changes in articular cartilage, subchondral trabecular bone (STB), and other articular tissues [3–5]. Previous studies suggested that STB is closely related to the structure and function of the covered cartilage, and they interact as a functioning synergistic unit [6–8]. Moreover, there is in vitro and in vivo evidence of biochemical and molecular crosstalk between cartilage and STB and STB microarchitecture changes in early-stage knee OA [9, 10]. In particular, the STB is a shock absorber that buffers the mechanical shock during joint movement, and its structural and property change affects the mechanical load exerted on the cartilage and may play a key role in the initiation and development of OA [4].

The properties and structure of the STB in OA could be characterized by dual X-ray absorptiometry (DXA) [11], X-ray computed tomography (CT) [12], CT arthrography [13], micro-CT (μCT) [14], and magnetic resonance imaging (MRI) [15, 16]. Previous studies have reported that knee OA severity, based on histological score [17], Kellgren-Lawrence (K-L) grade [18], cartilage defects, and cartilage thinning, was positively correlated with tibial plateau subchondral bone mineral density (BMD), trabecular bone volume fraction (BV/TV), trabecular number (Tb.N), and trabecular thickness (Tb.Th), suggesting that subchondral bone is closely related to OA severity.

The occurrence and deterioration of OA are widely known to result from local mechanical factors acting under systemic susceptibility [19]. Knee alignment, hip-knee-ankle (HKA) angle, as the frontal plane loading index, plays a crucial role in the load distribution of the medial and lateral tibiofemoral compartments. Knee malalignment causes the load-bearing axis to be biased to one side; therefore, the resulting moment arm increases the load on the side compartment, which is a significant risk factor for predicting the onset and progression of OA [20].

Aberrant knee load index has been associated with local variations in tibial periarticular BMD based on DXA [18, 21, 22]. However, DXA as a two-dimensional quantitative tool can neither distinguish cortical bone from trabecular bone nor characterize the bone microarchitecture. Thus, it is necessary to analyze STB microarchitecture, because better understanding of the effects of knee joint loading on local changes in STB microarchitecture can help us understand their roles in the development of knee OA. Roberts et al. [23] have shown a significant correlation between knee mechanical axis deviation (MAD) and tibial STB microarchitecture, and Finnila et al. [17] have shown a significant correlation between OARSI score and STB microarchitecture. However, to the best of our knowledge, no recent study has simultaneously evaluated the association between knee alignment, STB microarchitecture, and OA severity index of the corresponding compartment in the same patients. Data on this can help us understand more deeply the factors that affect the occurrence and development of OA and identify potential targets for diagnosis and surgical or non-invasive therapies of OA.

In this study, we aimed to investigate the relationship between STB microarchitecture and HKA angle and to explore the relationship between STB microarchitecture and OA severity under different HKA angles in end-stage knee OA patients. We hypothesized that the HKA angle is closely related to the microarchitecture variation of the STB of the medial and lateral tibial plateaus. Furthermore, we proposed that the variation in STB microarchitecture is related to OA severity.

## Methods

### Participants

Knee OA patients scheduled for total knee arthroplasty (TKA) were recruited from the orthopedics departments at the Ninth People’s Hospital, Shanghai Jiaotong University School of Medicine. The diagnosis of OA was based on the American College of Rheumatology criteria [24]. All patients undergoing surgery experienced knee pain and were not satisfied with the effects of noninvasive treatment. The K-L grading [25] of the whole knee joints indicated for surgery was 2 to 4. Patients excluded from this study were those with a history of inflammatory arthritis, had neurological disorders that would affect walking, had severe cardiovascular or pulmonary disease, and had isolated patellofemoral knee OA. This study received ethics approval from the Shanghai Ninth People’s Hospital Human Research Ethics Committees (No.2018-179-T137). Informed consent was obtained from all patients included in the study.

### Clinical and radiographic data measurement

**Fig. 1:**
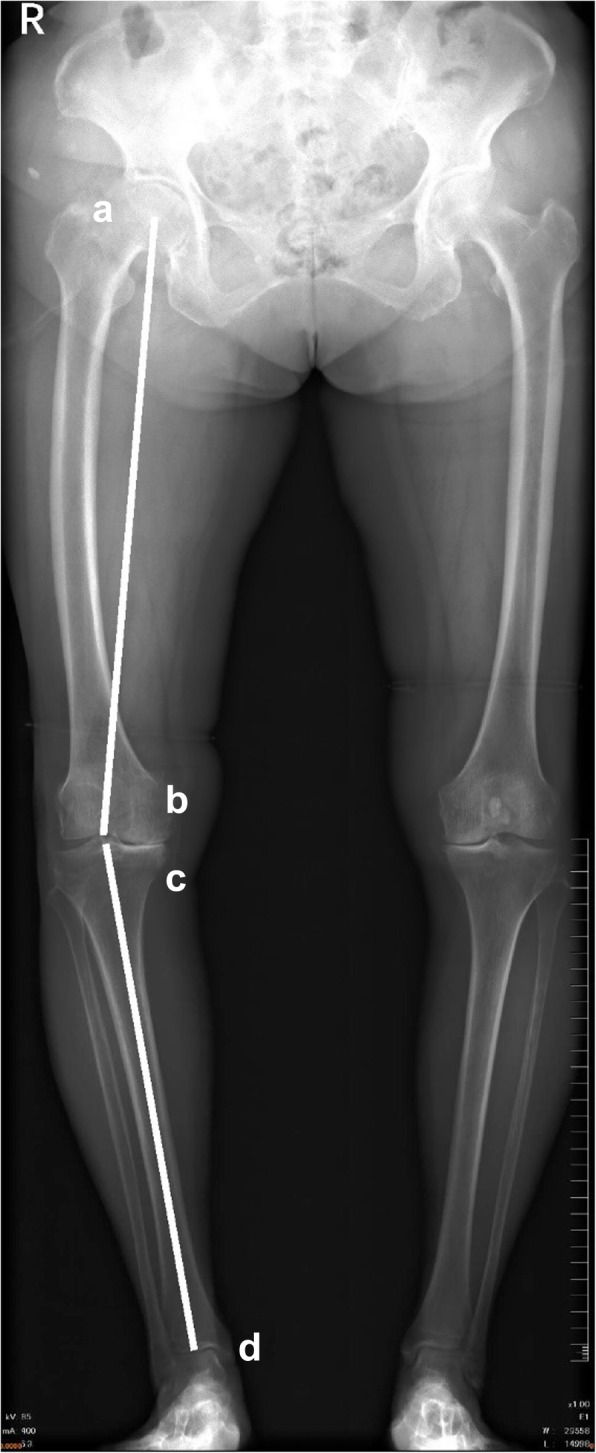
Measurement of knee alignment (hip-knee-ankle angle) based on full-leg standing anteroposterior radiographs. **a** The center of the femoral head. **b** The center of the femoral condyles. **c** The center of the tibial plateau. **d** The center of the superior surface of the talus

All patients underwent full-leg standing digital anteroposterior radiography preoperatively to measure the HKA angle and K-L grade [25] of the affected joint. Knee alignment was represented by the HKA angle formed between the mechanical axis of the femur and tibia (Fig. 1) [26]. All patients were divided into the varus alignment group (HKA angle > 0° in the varus direction) and valgus alignment group (HKA angle > 0° in the valgus direction), and positive values represent varus knee alignment [19, 27, 28]. Height, body mass, and Western Ontario and McMaster Universities Osteoarthritis Index (WOMAC) scores were derived from the patient’s medical records. A visual analog scale version of the WOMAC was completed by each patient to assess the status of knee OA [29].

### Micro-computed tomography scan and analysis

**Fig. 2:**
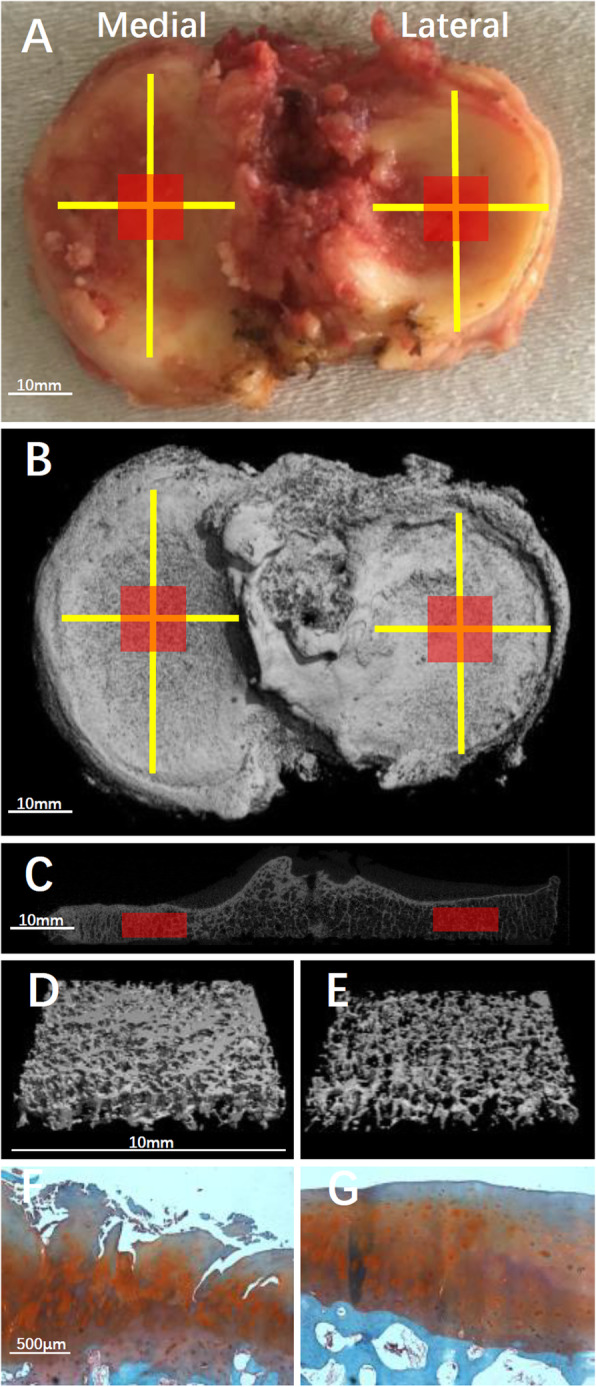
**a** Macroscopic, micro-CT and histological images of tibial plateaus from an excised right knee tibial plateau. Red squares represent the location of volumes of interest (VOIs) of the medial and lateral tibial plates. **b** 3D micro-CT image of the excised tibial plateau. **c** 2D coronal micro-CT transverse image of the tibial plateau. **d** Specimen from the medial tibial plateau showing high bone volume fraction (BV/TV = 51%). **e** Specimen from the lateral tibial plateau showing low bone volume fraction (BV/TV = 14%). **f** and **g** are histological photographs corresponding to **d** and **e**, respectively

Tibial plateaus (*n* = 71) were retrieved from TKA and scanned using a μCT scanner (μCT 80, Scanco Medical AG, Switzerland). Briefly, the following scanning parameters were used: 37 μm isotropic voxel size, 70 kV voltage, 114 μA current, and 300 ms integration time. The μCT system software (Image Processing Language, v4.29d, Scanco Medical AG, Bassersdorf, Switzerland) was used to process the scanned image data. Volumes of interest (VOI) of STB were selected for each medial and lateral tibial plateau separately. VOIs were determined first by locating the center of tibia plateau. Center was defined as the intersection of the sagittal axis corresponding to the maximum anterior and posterior lengths of the unilateral condyle and the coronal axis corresponding to the maximum width of the inside and outside of the condyle. Distance was measured using the software and did not include osteophytes. The VOI contained only STB and was identified as a cube with a cross-section of 10 × 10 mm and a thickness of 3.7 mm using the semiautomatic contouring method. The upper surface of the VOI was adjacent to the lower surface of the subchondral bone plate and extended 3.7 mm distally. The following STB microarchitecture parameters were measured for VOI: BV/TV, Tb.N, trabecular separation (Tb.Sp), Tb.Th, and specific bone surface (BS/BV). The ratios of STB microarchitecture parameters to medial and lateral plateaus (medial-to-lateral, M:L) were then computed (Fig. 2).

### Histology

After a μCT scan, tissue plugs corresponding to the VOI of tibial plateaus were processed for histological analysis. The condyle midpoint and VOI range were determined using the scale according to their definition in the software. Paraffin-embedded decalcified tissue was sectioned to 5 mm sections and stained with Safranin O and Fast Green for Osteoarthritis Research Society International (OARSI) scoring. Three sagittal longitudinal tissue sections through the medial, lateral, and center of each tissue plug were scored by three independent evaluators. The average score from three evaluators was used as the final OARSI score for further analyses. The evaluators were blinded with respect to the HKA angle, grouping, and macro-description of the specimen [30] (Fig. 2).

### Statistics

*T* test, *χ*^2^ tests, and Mann–Whitney *U* tests were used to compare differences in means and proportions of patients characteristics as appropriate. The linear relationships of STB microarchitecture parameters (of each tibial plateau and medial and lateral tibial plateaus ratio), HKA angle, and OA severity (OARSI score and K-L grade) in all patients, varus alignment group, and valgus alignment group, respectively, were examined using Pearson’s or Spearman’s correlations based on the normality (Shapiro–Wilks test) of data. The STB microarchitecture parameters of the medial and lateral tibial plateau were compared between varus and valgus alignment groups using independent-sample *t* test or Satterthwaite *t* test according to the homogeneity of variance (Levene’s test) of the data. The STB microarchitecture parameters of medial and lateral tibial plateaus were compared using paired *t* test in the varus alignment group and valgus alignment group, respectively. We used hierarchical multiple linear regression analyses to explain the variance in the STB microarchitecture and selected age, sex, and body mass index (BMI) as covariates for our base model to evaluate the relationships between the HKA angle and STB microarchitecture. We assessed multicollinearity between all independent variables in each model using variance inflation factor. We report adjusted *R*^2^, change in *R*^2^ from the base model (Δ*R*^2^), and *p* values. The significance level was set to *p* < 0.05. Statistical analysis was performed using SPSS Statistics 22 (IBM Corp., Armonk, NY, USA).

## Results

Seventy-one knee OA patients scheduled for TKA were included in this study. The physical characteristics and radiographic features of patients are reported in Table 1.

**Table 1:** Demographic and clinical characteristics of total knee arthroplasty patients

Characteristic	All	Varus	Valgus	*p*
	(*n* = 71)	(*n* = 60)	(*n* = 11)	
Age (years)	70 ± 8	70 ± 8	67 ± 8	0.330
Gender (male:females)	16:55	13:47	3:8	0.702
Affected limb (right:left)	30:41	25:35	5:6	1.000
Height (m)	1.62 ± 0.06	1.62 ± 0.07	1.63 ± 0.06	0.523
Body mass (kg)	67.1 ± 8.7	67.1 ± 9.2	66.7 ± 5.3	0.884
BMI (kg/m^2^)	25.5 ± 2.9	25.6 ± 2.9	25.1 ± 2.6	0.621
WOMAC	68 ± 29	67 ± 30	73 ± 21	0.518
Hip-knee-ankle angle (°)	7.5 ± 8.5	10.3 ± 5.3	− 7.9 ± 5.7	**< 0.001**
K-L grade (2:3:4)	2:35:34	2:27:31	0:8:3	0.189

Data are presented as either number or mean ± standard deviation

Significant values are in bold (*P* < 0.05)

*BMI* bone mass index, *K-L* Kellgren-Lawrence, *WOMAC* Western Ontario and McMaster Universities Arthritis Index

### Relationships between HKA angle and subchondral trabecular bone microarchitecture in the entire OA cohort

**Fig. 3:**
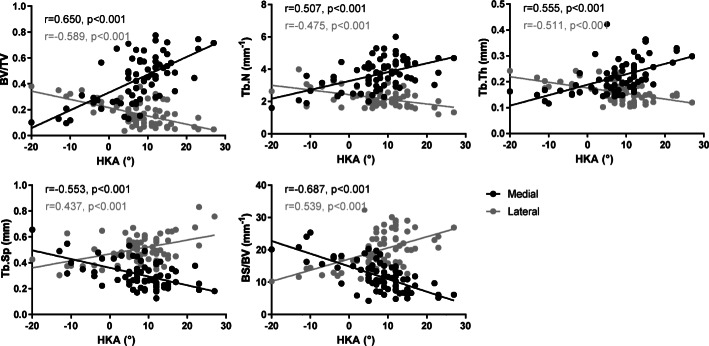
Scatter plot for Pearson’s correlations between hip-knee-ankle angle and subchondral trabecular bone microarchitecture parameters. BV/TV, bone volume fraction; Tb.N, trabecular number; Tb.Th, trabecular thickness; Tb.Sp, trabecular separation; BS/BV, specific bone surface

In all patient analysis, the HKA angle was significantly correlated with all tibial plateau STB microarchitecture parameters (Fig. 3, Table 2). The HKA angle had higher correlation with the M:L STB microarchitecture parameters than the absolute measurements of the medial or lateral, positively with M:L BV/TV, M:L Tb.N, and M:L Tb.Th, and negatively with M:L Tb.Sp and M:L BS/BV. In addition, the correlation between the HKA angle and STB microarchitecture parameters of the medial tibial plateau was higher than that of the lateral tibial plateau, the highest of which were BV/TV (*r* = 0.650, CI 0.492, 0.763) and BS/BV (*r* = − 0.687, 95% CI − 0.788, − 0.543). These findings indicated that the STB of the affected side tibial plateau increased in bone volume, trabecular number, and trabecular thickness and decreased in trabecular separation with the aggravation of the knee alignment deviation.

**Table 2:** Relationships between knee alignment (hip-knee-ankle angle, HKA angle) and subchondral trabecular bone microarchitecture parameters

Paramaters	Medial	Lateral	M:L
BV/TV	0.650 (0.492, 0.763)**	− 0.589 (− 0.745, − 0.389)**	0.663 (0.538, 0.765)**
Tb.N	0.507 (0.305, 0.672)**	− 0.475 (− 0.675, − 0.214)**	0.619 (0.404, 0.778)**
Tb.Th	0.555 (0.373, 0.703)**	− 0.511 (− 0.659, − 0.325) **	0.660 (0.538, 0.763)**
Tb.Sp	− 0.553 (− 0.717, − 0.310)**	0.437 (0.179, 0.635)**	− 0.679 (− 0.882, − 0.449)**
BS/BV	− 0.687 (− 0.788, − 0.543)**	0.539 (0.374, 0.671)**	− 0.824 (− 0.896, − 0.697)**

The indicated values are Pearson’s coefficient and 95% confidence interval in brackets

*M:L* medial-to-lateral ratio, *BV/TV* bone volume fraction, *Tb.N* trabecular number, *Tb.Th* trabecular thickness, *Tb.Sp* trabecular separation, *BS/BV* specific bone surface

**P < 0.01

### Contribution of age, sex, BMI, and HKA angle to variation in the subchondral trabecular bone microarchitecture

No evidence of multicollinearity was found between independent variables in any of our models. The HKA angle was entered in all regression models for prediction of medial tibial plateau STB microarchitecture, after controlling for age, sex, and BMI (Table 3). After adding the HKA angle to the base model (age, sex, and BMI), the coefficient of determination (*R*^2^) for STB microarchitecture parameters all significantly improved with Δ*R*^2^ (BV/TV) = 0.326, Δ*R*^2^ (Tb.N) = 0.193, Δ*R*^2^ (Tb.Th) = 0.246, Δ*R*^2^ (Tb.Sp) = 0.231, and Δ*R*^2^ (BS/BS) = 0.369 in medial tibial plateau and with Δ*R*^2^ (BV/TV) = 0.345, Δ*R*^2^ (Tb.N) = 0.228, Δ*R*^2^ (Tb.Th) = 0.243, Δ*R*^2^ (Tb.Sp) = 0.189, and Δ*R*^2^ (BS/BS) = 0.272 in lateral tibial plateau. These showed that the HKA angle could explain the additional variation in all five STB microarchitecture parameters, when controlled for age, sex, and BMI.

**Table 3:** Summary of multiple linear regression analysis, for prediction of medial and lateral tibial plateau subchondral trabecular bone microarchitecture parameters

Dependent variables	Model	*R*^2^ (*p* value)	Δ*R*^2^ (*p* value)
Medial			
BV/TV	Age, sex, BMI	**0.182 (0.004)**	
	Age, sex, BMI, HKA angle	**0.509 (< 0.001)**	**0.326 (< 0.001)**
Tb.N	Age, sex, BMI	**0.213 (0.001)**	
	Age, sex, BMI, HKA angle	**0.407 (< 0.001)**	**0.193 (< 0.001)**
Tb.Th	Age, sex, BMI	0.103 (0.062)	
	Age, sex, BMI, HKA angle	**0.349 (< 0.001)**	**0.246 (< 0.001)**
Tb.Sp	Age, sex, BMI	**0.176 (0.004)**	
	Age, sex, BMI, HKA angle	**0.407 (< 0.001)**	**0.231 (< 0.001)**
BS/BV	Age, sex, BMI	**0.174 (0.005)**	
	Age, sex, BMI, HKA angle	**0.543 (< 0.001)**	**0.369 (< 0.001)**
Lateral			
BV/TV	Age, sex, BMI	**0.108 (0.014)**	
	Age, sex, BMI, HKA angle	**0.491 (< 0.001)**	**0.345 (< 0.001)**
Tb.N	Age, sex, BMI	**0.121 (0.034)**	
	Age, sex, BMI, HKA angle	**0.349 (< 0.001)**	**0.228 (< 0.001)**
Tb.Th	Age, sex, BMI	0.092 (0.089)	
	Age, sex, BMI, HKA angle	**0.335 (< 0.001)**	**0.243 (< 0.001)**
Tb.Sp	Age, sex, BMI	**0.177 (0.004)**	
	Age, sex, BMI, HKA angle	**0.366 (< 0.001)**	**0.189 (< 0.001)**
BS/BV	Age, sex, BMI	0.102 (0.064)	
	Age, sex, BMI, HKA angle	**0.374 (< 0.001)**	**0.272 (< 0.001)**

Significant values are in bold

*BMI* body mass index, *BV/TV* bone volume fraction, *Tb.N* trabecular number, *Tb.Th* trabecular thickness, *Tb.Sp* trabecular separation, *BS/BV* specific bone surface

### Relationships between OA severity and subchondral trabecular bone microarchitecture and HKA angle in the entire OA cohort

In the entire OA cohort, the HKA angle and all five STB microarchitecture parameters were significantly correlated with the OARSI score in both medial and lateral tibia plateaus (*p* < 0.01). The correlation between the HKA angle and medial tibial plateau OARSI score (*r* = 0.792, 95% CI 0.683, 0.862) was higher than that of the lateral tibial plateau (*r* = − 0.365, 95% CI − 0.550, − 0.123). Regardless of the medial or lateral tibia plateau, BV/TV, Tb.N, and Tb.Th were positively correlated with the OARSI score, while Tb.Sp and BS/BV were negatively correlated with the OARSI score. These results suggested that the more severe cartilage degeneration corresponds to the greater bone volume of the STB, and the denser and thicker STB, and the smaller trabecular separation, and the more severe deviation of knee alignment (Table 4).

**Table 4:** Relationships between subchondral trabecular bone microarchitecture parameters and OA severity index

Parameters	OARSI score		K-L grade	
	Medial	Lateral	Medial	Lateral
**HKA**	0.792 (0.683, 0.862)**	− 0.365 (− 0.550, − 0.123)**	0.743 (0.616, 0.831)**	− 0.325 (− 0.543, − 0.097)**
**BV/TV**	0.853 (0.782, 0.913)**	0.754 (0.643, 0.837)**	0.742 (0.608, 0.852)**	0.674 (0.519, 0.789)**
**Tb.N**	0.607 (0.464, 0.728)**	0.625 (0.486, 0.755)**	0.588 (0.397, 0.734)**	0.570 (0.388, 0.711)**
**Tb.Th**	0.743 (0.642, 0.828)**	0.599 (0.434, 0.743)**	0.651 (0.501, 0.770)**	0.518 (0.320, 0.678)**
**Tb.Sp**	− 0.649 (− 0.752, − 0.520)**	− 0.625 (− 0.739, − 0.497)**	− 0.574 (− 0.716, − 0.385)**	− 0.560 (− 0.705, − 0.379)**
**BS/BV**	− 0.840 (− 0.894, − 0.767)**	− 0.705 (− 0.811, − 0.577)**	− 0.713 (− 0.819, − 0.573)**	− 0.579 (− 0.724, − 0.392)**

The indicated values are Pearson’s or Spearman’s coefficient and 95% confidence interval in brackets

*BV/TV* bone volume fraction, *Tb.N* trabecular number, *Tb.Th* trabecular thickness, *Tb.Sp* trabecular separation, *BS/BV* specific bone surface

**P < 0.01

### Relationships between subchondral trabecular bone microarchitecture, HKA angle, and OA severity in the varus and valgus alignment groups

After stratifying patients based on the HKA angle, the correlation between the HKA angle and STB microarchitecture was found to be the same as the trend of the overall analysis. In the varus alignment group, all STB microarchitecture parameters, except Tb.Th (*r* = − 0.225, 95% CI − 0.412, − 0.016) of the lateral tibial plateau, were significantly correlated with the HKA angle. In addition, the HKA angle had higher correlation with the M:L STB microarchitecture parameters than with the absolute measurements of the medial or lateral. In the valgus alignment group, only BV/TV was significantly correlated with the HKA angle regardless of the medial tibial plateau, lateral tibial plateau, or M:L STB microarchitecture parameters. In addition, Tb.N and Tb.Sp in the medial tibial plateau and the M:L Tb.Th and M:L BS/BV were significantly correlated with the HKA angle. From the above results, we can find that the correlation between BV/TV and HKA angle is the most stable (Table 5).

**Table 5:** Relationships between knee alignment (hip-knee-ankle angle) and subchondral trabecular bone microarchitecture parameters for varus and valgus alignment group

Paramaters	Medial	Lateral	M:L
Varus (*n* = 60)			
BV/TV	0.466 (0.200, 0.654)**	− 0.277 (− 0.462, − 0.048)*	0.628 (0.357, 0.776)**
Tb.N	0.295 (0.079, 0.503)*	− 0.291 (− 0.511, 0.021)*	0.539 (0.220, 0.733)**
Tb.Th	0.432 (0.153, 0.661)**	− 0.225 (− 0.412, − 0.016)	0.503 (0.298, 0.659)**
Tb.Sp	− 0.332 (− 0.511, − 0.094)**	0.389 (0.019, 0.609)**	− 0.459 (− 0.635, − 0.223)**
BS/BV	− 0.479 (− 0.632, − 0.255)**	0.282 (0.068, 0.475)*	− 0.530 (− 0.664, − 0.323)**
Valgus (*n* = 11)			
BV/TV	0.685 (0.520, 0.893)*	− 0.645 (− 0.924, − 0.040)*	0.674 (0.443, 0.888)*
Tb.N	0.616 (− 0.072, 0.927)*	− 0.243 (− 0.796, − 0.347)	0.428 (− 0.245, 0.815)
Tb.Th	0.399 (− 0.142, 0.770)	− 0.315 (− 0.917, − 0.569)	0.733 (0.476, 0.906)*
Tb.Sp	− 0.696 (− 0.955, 0.057)*	0.223 (− 0.345, 0.756)	− 0.590 (− 0.903, 0.105)
BS/BV	− 0.556 (− 0.823, − 0.250)	0.507 (− 0.304, 0.867)	− 0.841 (− 0.964, − 0.486)**

The indicated values are Pearson’s or Spearman’s coefficient and 95% confidence interval in brackets

*M:L* medial-to-lateral ratio, *BV/TV* bone volume fraction, *Tb.N* trabecular number, *Tb.Th* trabecular thickness, *Tb.Sp* trabecular separation, *BS/BV* specific bone surface

*P < 0.05, **P < 0.01

The relationships between STB microarchitecture parameters and OA severity index for varus and valgus alignment groups were shown in Table 6. In the varus alignment group, a significant correlation was found between the STB microarchitecture, HKA angle, and OARSI score (*p* < 0.01), and the trend was the same as that in the overall analysis. The HKA angle was significantly correlated with the OARSI of the medial and lateral tibial plateaus. Among all the five STB microarchitecture parameters, the correlation between BV/TV and OARSI score was the strongest, regardless of medial (*r* = 0.828, 95% CI 0.722, 0.905) or lateral (*r* = 0.811, 95% CI 0.729, 0.875) tibial plateau. In the valgus alignment group, the HKA angle was significantly correlated with the OARSI of the medial and lateral tibial plateaus. Among the five STB microarchitecture parameters, BV/TV, Tb.Th, and BS/BV of the medial tibial plateau and BV/TV of the lateral tibial plateau were significantly correlated with the OARSI score (Table 6).

**Table 6:** Relationships between subchondral trabecular bone microarchitecture parameters and OA severity index for varus and valgus alignment group

Parameters	OARSI score		K-L grade	
	M	L	M	L
**Varus (*n*** **= 60)**				
**HKA**	0.574 (0.338, 0.719)**	− 0.337 (− 0.530, − 0.085)**	0.664 (0.494, 0.782)**	− 0.088 (− 0.334, 0.147)
**BV/TV**	0.828 (0.722, 0.905)**	0.811 (0.729, 0.875)**	0.680 (0.492, 0.816)**	0.618 (0.455, 0.750)**
**Tb.N**	0.514 (0.306, 0.680)**	0.722 (0.598, 0.812)**	0.518 (0.301, 0.700)**	0.621 (0.474, 0.745)**
**Tb.Th**	0.700 (0.553, 0.807)**	0.688 (0.555, 0.789)**	0.565 (0.356, 0.720)**	0.443 (0.234, 0.623)**
**Tb.Sp**	− 0.594 (− 0.725, − 0.410)**	− 0.643 (− 0.775, − 0.471)**	− 0.506 (− 0.697, − 0.281)**	− 0.589 (− 0.726, − 0.431)**
**BS/BV**	− 0.775 (− 0.846, − 0.672)**	− 0.777 (− 0.857, − 0.668)**	− 0.638 (− 0.783, − 0.445)**	− 0.515 (− 0.685, − 0.309)**
**Valgus (*n*** **= 11)**				
**HKA**	0.697 (0.409, 0.963)*	− 0.718 (− 0.923, − 0.236)*	0.662 (0.177, 0.901)*	− 0.504 (− 0.835, 0.054)
**BV/TV**	0.661 (0.184, 0.909)*	0.749 (0.388, 0.922)**	0.777 (0.407, 0.889)**	0.429 (− 0.131, 0.793)
**Tb.N**	0.417 (− 0.205, 0.808)	0.314 (− 0.189, 0.761)	0.299 (− 0.425, 0.843)	− 0.143 (− 0.678, 0.370)
**Tb.Th**	0.680 (0.097, 0.917)*	− 0.005 (− 0.716, 0.638)	0.299 (− 0.472, 0.848)	− 0.048 (− 0.687, 0.682)
**Tb.Sp**	− 0.397 (− 0.781, − 0.198)	− 0.463 (− 0.797, − 0.007)	− 0.359 (− 0.843, − 0.334)	− 0.095 (− 0.461, 0.379)
**BS/BV**	− 0.702 (− 0.927, − 0.143)*	− 0.232 (− 0.698, 0.471)	− 0.538 (− 0.878, − 0.121)	0.048 (− 0.676, 0.687)

The indicated values are Pearson’s or Spearman’s coefficient and 95% confidence interval in brackets

*HKA* hip-knee-ankle angle, *BV/TV* bone volume fraction, *Tb.N* trabecular number, *Tb.Th* trabecular thickness, *Tb.Sp* trabecular separation, *BS/BV* specific bone surface

*P < 0.05, **P < 0.01

### Comparison of subchondral trabecular bone microarchitecture between knee alignment groups based on the HKA angle

**Fig. 4:**
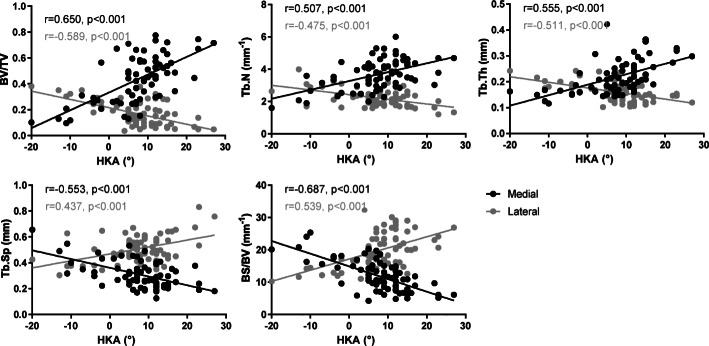
Comparisons of subchondral trabecular bone microarchitecture parameters among knee alignment (hip-knee-ankle angle, HKA angle) groups. BV/TV, bone volume fraction; Tb.N, trabecular number; Tb.Th, trabecular thickness; Tb.Sp, trabecular separation; BS/BV, specific bone surface. Significant differences are indicated by brackets (*p* < 0.05)

In the varus alignment group, BV/TV, Tb.N, and Tb.Th were significantly larger and Tb.Sp and BS/BV were significantly smaller in the medial tibial plateau than in the lateral tibial plateau. In the valgus alignment group, Tb.Th was significantly larger in the lateral tibial plateau and BS/BV significantly smaller than in the medial tibial plateau. Other parameters were not statistically different between the medial and lateral tibial plateaus. In the medial tibia plateau, BV/TV, Tb.N, and Tb.Th were significantly larger in the varus alignment group and Tb.Sp and BS/BV were significantly larger than in the valgus alignment group, and the lateral tibia plateau had the opposite results (Fig. 4).

## Discussion

This study investigated the variation in tibial plateau STB microarchitecture in end-stage knee OA patients and its association with OA severity under the difference of knee alignment. Tibial plateau STB microarchitecture is associated with the HKA angle and OA severity. With the increase in varus angle and OA severity, the STB in the medial tibia plateau increased in bone volume, trabecular number, and trabecular thickness and decreased in trabecular separation.

With regard to the relationship of knee alignment and subchondral bone, the HKA angle and the ratio of M:L subchondral bone surface area on the tibia and femur are significantly correlated, which suggested that the subchondral bone could change adaptively under the influence of knee alignment [31]. Several previous studies have also found a correlation between knee load and proximal tibial BMD based on DXA [18, 21, 22]. However, as a two-dimensional imaging technology, DXA can neither distinguish trabecular bone from cortical bone for analysis alone nor can characterize STB microarchitecture, which has been shown to change under OA [32, 33]. Thus, it is necessary to study the changes of the STB microarchitecture under OA to understand its effect on OA. MRI was used to evaluate STB microarchitecture in previous studies, but its limited spatial resolution (0.2 × 0.2 × 1.0 mm) limits its ability in microarchitecture analysis [15, 22].

A recent study on the relationship between knee loading index and tibial STB microarchitecture (using μCT) had similar results to that reported in the current finding [23]. The Pearson’s correlation coefficient of MAD with M:L BV/TV in that study was 0.74 (*p* < 0.01), which is comparable with that of the HKA angle and M:L BV/TV in our study (*r* = 0.66, *p* < 0.01). In the multiple regression analysis of the current study, the HKA angle could explain the additional variation in all five STB microarchitecture parameters (Table 3), when controlled for age, sex, and BMI, which are parameters that may influence tibial STB microarchitecture [34]. In addition, our study found that the M:L ratios of the STB microarchitecture parameters had a stronger correlation with the HKA angle than the absolute measurements of the medial and lateral tibial plateaus, which supported the idea that the HKA angle is a coronal load distribution indicator that simultaneously affects the load distribution of the medial and lateral compartments in the knee joint. This finding is in agreement with a previous study that has shown that the correlation between the HKA angle and BMD of the M:L ratios in the tibia is stronger than that of absolute measurement of the unilateral tibia [22].

Previous studies have explored the effect of knee alignment changes on the subchondral bone by analyzing BMD changes of the subchondral bone before and after undergoing high tibial osteotomy, a surgery for correction of knee malalignment [35, 36]. The results showed that following varus deformity correcting, the M:L ratio of the subchondral bone density decreases. However, these studies lacked a control group. In the future, larger randomized controlled studies are necessary to determine whether these interventions directly acting on the knee alignment can alter the subchondral bone BMD and STB microarchitecture. This can be done based on high-resolution peripheral quantitative CT (HR-pQCT) imaging systems, which permit examination of knee periarticular STB microarchitecture in vivo [37, 38].

In addition, our study explored the changes in the STB microarchitecture of the medial and lateral tibia in different HKA angle groups. In the varus alignment group, BV/TV, Tb.N, and Tb.Th were significantly larger and Tb.Sp and BS/BV were significantly smaller in the medial tibial plateau than in the lateral tibial plateau. These findings prove once again the correlation between knee HKA angle and STB microarchitecture. For the intra-group comparison of the STB of the medial and lateral tibia plateaus, significant differences were noted in all five STB microarchitecture parameters between the medial and lateral tibia in the varus alignment group, which indicates that the STB of the medial tibia has suffered from excessive load and had a more serious sclerosis change under the more severe varus alignment deviation in the knee; however, this was not observed in the valgus alignment group. These findings are similar to those of a previous study that analyzed the relationship between knee alignment and tibial microarchitecture, suggesting that knee alignment affects the load distribution on the medial and lateral tibia, thereby altering its STB microarchitecture [39]. These findings suggest that mechanical load is more distributed in the medial compartment in the normally aligned knee and varus alignment deviation further increases the stress load on the medial compartment. Valgus alignment deviation increases the load distribution on the lateral compartment; however, more load is still distributed in the medial compartment until the valgus is large enough [40, 41].

The relationship between subchondral bone degeneration and OA severity has previously been reported in several studies. Among them, Omoumi et al. [13] have shown that in knee OA, cartilage thickness and subchondral bone mineral density based on CT arthrography are negatively correlated, which indicate mutual adaptation in cartilage-subchondral bone loses in the OA state. Bobinac et al. [33] showed the same trends as reported in the current study in subchondral bone and cartilage degeneration under OA; however, they used a 2D histology method for STB microarchitecture and did not consider the effect of knee alignment changes. Finnila et al. [17] showed that the STB microarchitecture parameters based on micro-CT were highly correlated with OARSI scores of cartilage degeneration, indicating that bone sclerosis and cartilage degeneration are coupled. In the present study, we found that cartilage degeneration is significantly associated with more severe sclerosis changes in STB microarchitecture, which supports the theory of a subchondral bone-cartilage functional unit where the OA disease state could destroy the homeostatic relationship between them under abnormal knee loads. In addition, Bhatla et al. [42] showed that subchondral bone changes may be indicative of early OA pathogenesis of post-traumatic knee injuries, and Chen et al. [43] also showed that abnormal STB remodeling is earlier than that of cartilage change and may contribute to the early pathogenesis of T2D-associated knee OA. However, given the cross-sectional design of the present study, we cannot prove the sequence of occurrence and causality between cartilage and STB, which requires further research to investigate the role of STB in progression of OA.

As knee OA with varus and valgus may represent distinct disease phenotypes [44], it is necessary to investigate the correlation between the HKA angle, OA severity, and STB microarchitecture in the varus and valgus alignment subgroups, respectively. In the varus alignment subgroup, associations between the HKA angle and M:L BV/TV were comparable (*r* = 0.628 [− 0357, 0.776], *p* < 0.001) to that reported in scientific literatures between the HKA angle and M:L BMD (*r* range 0.44–0.53) [18, 21]. Although the sample size of the valgus alignment group is limited (*n* = 11), the HKA angle is also significantly correlated with M:L BV/TV(*r* = 0.628 [− 0357, 0.776], *p* = 0.023). To the best of our knowledge, this is the first report on the significant correlation between knee loading index and STB microarchitecture parameters in valgus knee alignment cohort. Therefore, current studies have shown a significant correlation between the HKA angle and M:L BV/TV, regardless of varus or valgus knee alignment. In the valgus alignment group, the correlation between the STB microarchitecture and OARSI score was less significant than that in the varus alignment group, possibly due to the limited sample size.

Several limitations of this study should be discussed. First, we only investigated the STB microarchitecture of the tibial plateau, while the medial and lateral femoral condyles as another part of the tibiofemoral joint also reflected the degeneration of the knee joint under different load conditions. Future research is necessary to add the measurement of femoral condyle STB microarchitecture to the above analysis. Second, because μCT can only be used to analyze human tissue samples in vitro, the samples in this study were limited to patients with TKA. As we know, the progression of OA and the wear of cartilage are the reasons for knee alignment deviation. And we do not have normal, non-osteoarthritis tibial plateau specimens as controls. Hence, we could not determine whether the relationship between the HKA angle and STB microarchitecture shown in this study also exists in patients before TKA or can reflect the early OA disease state. Fortunately, HR-pQCT has been used to evaluate human knee periarticular STB microarchitecture in vivo, which could examine the above relationships in early-stage OA and nonpathological knee. Third, given the cross-sectional design of this study, we were unable to determine the causal directionality of the relationship between OA severity, HKA angle, and STB microarchitecture. A longitudinal study based on HR-pQCT is necessary to investigate the cause of the association of knee alignment with STB microarchitecture. Finally, only 11 patients were included in the valgus alignment group in the current study. The small sample size may influence the significance of the test results after grouping.

## Conclusion

In summary, this study found that tibial plateau STB microarchitecture of end-stage knee OA patients is associated with the HKA angle and OA severity. With the increase of knee alignment deviation and OA severity, the STB of the affected side tibial plateau increased in bone volume, trabecular number, and trabecular thickness and decreased in trabecular separation, which suggests that knee malalignment may promote abnormal STB remodeling by altering joint load distribution, thereby affecting the progression of OA. These findings may contribute to a better understanding of the effects of knee joint loading on local changes in STB microarchitecture and the role of both in the development of knee OA. In addition, the influence of knee alignment should be considered in the future study of knee periarticular bone structure and properties. Future work that elucidates the cause of the relationship between joint loading and STB microarchitectural changes to identify new targets for OA therapies is required.

## Data Availability

The datasets used and/or analyzed during the current study are available from the corresponding author on reasonable request.

## Abbreviations

OA: Osteoarthritis
STB: Subchondral trabecular bone
HKA angle: Hip-knee-ankle angle
TKA: Total knee arthroplasty
DXA: Dual X-ray absorptiometry
μCT: Micro-CT
MRI: Magnetic resonance imaging
K-L grade: Kellgren-Lawrence grade
BMD: Bone mineral density
BV/TV: Bone volume fraction
Tb.N: Trabecular number
Tb.Th: Trabecular thickness
MAD: Mechanical axis deviation
WOMAC: Western Ontario and McMaster Universities Osteoarthritis Index
VOI: Volumes of interest
M:L: Medial-to-lateral
OARSI: Osteoarthritis Research Society International scoring
HR-pQCT: High-resolution peripheral quantitative CT

## References

[1] Glyn-Jones S, Palmer AJ, Agricola R, Price AJ, Vincent TL, Weinans H (2015). Osteoarthritis. Lancet.

[2] Cross M, Smith E, Hoy D, Nolte S, Ackerman I, Fransen M (2014). The global burden of hip and knee osteoarthritis: estimates from the global burden of disease 2010 study. Ann Rheum Dis.

[3] Loeser RF, Goldring SR, Scanzello CR, Goldring MB (2012). Osteoarthritis: a disease of the joint as an organ. Arthritis Rheum.

[4] Lories RJ, Luyten FP (2011). The bone-cartilage unit in osteoarthritis. Nat Rev Rheumatol.

[5] Gallo J, Raska M, Kriegova E, Goodman SB (2017). Inflammation and its resolution and the musculoskeletal system. J Orthop Transl.

[6] Burr DB, Gallant MA (2012). Bone remodelling in osteoarthritis. Nat Rev Rheumatol.

[7] Imhof H, Sulzbacher I, Grampp S, Czerny C, Youssefzadeh S, Kainberger F (2000). Subchondral bone and cartilage disease: a rediscovered functional unit. Investig Radiol.

[8] Zhen G, Wen C, Jia X, Li Y, Crane JL, Mears SC (2013). Inhibition of TGF-beta signaling in mesenchymal stem cells of subchondral bone attenuates osteoarthritis. Nat Med.

[9] Yuan XL, Meng HY, Wang YC, Peng J, Guo QY, Wang AY (2014). Bone-cartilage interface crosstalk in osteoarthritis: potential pathways and future therapeutic strategies. Osteoarthr Cartil.

[10] Kroker A, Bhatla JL, Emery CA, Manske SL, Boyd SK (2018). Subchondral bone microarchitecture in ACL reconstructed knees of young women: a comparison with contralateral and uninjured control knees. Bone..

[11] Dore D, Quinn S, Ding C, Winzenberg T, Jones G (2009). Correlates of subchondral BMD: a cross-sectional study. J Bone Miner Res.

[12] Burnett WD, Kontulainen SA, McLennan CE, Hazel D, Talmo C, Wilson DR (2019). Knee osteoarthritis patients with more subchondral cysts have altered tibial subchondral bone mineral density. BMC Musculoskelet Disord.

[13] Omoumi P, Babel H, Jolles BM, Favre J (2019). Relationships between cartilage thickness and subchondral bone mineral density in non-osteoarthritic and severely osteoarthritic knees: in vivo concomitant 3D analysis using CT arthrography. Osteoarthr Cartil.

[14] Gatenholm B, Lindahl C, Brittberg M, Stadelmann VA (2019). Spatially matching morphometric assessment of cartilage and subchondral bone in osteoarthritic human knee joint with micro-computed tomography. Bone..

[15] Schneider E, Lo GH, Sloane G, Fanella L, Hunter DJ, Eaton CB (2011). Magnetic resonance imaging evaluation of weight-bearing subchondral trabecular bone in the knee. Skelet Radiol.

[16] Beuf O, Ghosh S, Newitt DC, Link TM, Steinbach L, Ries M (2002). Magnetic resonance imaging of normal and osteoarthritic trabecular bone structure in the human knee. Arthritis Rheum.

[17] Finnila MAJ, Thevenot J, Aho OM, Tiitu V, Rautiainen J, Kauppinen S (2017). Association between subchondral bone structure and osteoarthritis histopathological grade. J Orthop Res.

[18] Wada M, Maezawa Y, Baba H, Shimada S, Sasaki S, Nose Y (2001). Relationships among bone mineral densities, static alignment and dynamic load in patients with medial compartment knee osteoarthritis. Rheumatology.

[19] Sharma L, Song J, Felson DT, Cahue S, Shamiyeh E, Dunlop DD (2001). The role of knee alignment in disease progression and functional decline in knee osteoarthritis. JAMA..

[20] Sharma L, Song J, Dunlop D, Felson D, Lewis CE, Segal N (2010). Varus and valgus alignment and incident and progressive knee osteoarthritis. Ann Rheum Dis.

[21] Thorp LE, Wimmer MA, Block JA, Moisio KC, Shott S, Goker B (2006). Bone mineral density in the proximal tibia varies as a function of static alignment and knee adduction angular momentum in individuals with medial knee osteoarthritis. Bone..

[22] Lo GH, Merchant MG, Driban JB, Duryea J, Price LL, Eaton CB (2018). Knee alignment is quantitatively related to periarticular bone morphometry and density, especially in patients with osteoarthritis. Arthritis Rheum.

[23] Roberts BC, Solomon LB, Mercer G, Reynolds KJ, Thewlis D, Perilli E (2018). Relationships between in vivo dynamic knee joint loading, static alignment and tibial subchondral bone microarchitecture in end-stage knee osteoarthritis. Osteoarthr Cartil.

[24] Altman R, Asch E, Bloch D, Bole G, Borenstein D, Brandt K (1986). Development of criteria for the classification and reporting of osteoarthritis. Classification of osteoarthritis of the knee. Diagnostic and Therapeutic Criteria Committee of the American Rheumatism Association. Arthritis Rheum.

[25] Kellgren JH, Lawrence JS (1957). Radiological assessment of osteo-arthrosis. Ann Rheum Dis.

[26] Xie K, Jiang X, Han X, Ai S, Qu X, Yan M (2018). Association between knee malalignment and ankle degeneration in patients with end-stage knee osteoarthritis. J Arthroplast.

[27] Cooke TD, Sled EA, Scudamore RA (2007). Frontal plane knee alignment: a call for standardized measurement. J Rheumatol.

[28] Issa SN, Dunlop D, Chang A, Song J, Prasad PV, Guermazi A (2007). Full-limb and knee radiography assessments of varus-valgus alignment and their relationship to osteoarthritis disease features by magnetic resonance imaging. Arthritis Rheum.

[29] Bellamy N, Buchanan WW, Goldsmith CH, Campbell J, Stitt LW (1988). Validation study of WOMAC: a health status instrument for measuring clinically important patient relevant outcomes to antirheumatic drug therapy in patients with osteoarthritis of the hip or knee. J Rheumatol.

[30] Pritzker KP, Gay S, Jimenez SA, Ostergaard K, Pelletier JP, Revell PA (2006). Osteoarthritis cartilage histopathology: grading and staging. Osteoarthr Cartil.

[31] Eckstein F, Hudelmaier M, Cahue S, Marshall M, Sharma L (2009). Medial-to-lateral ratio of tibiofemoral subchondral bone area is adapted to alignment and mechanical load. Calcif Tissue Int.

[32] Patel V, Issever AS, Burghardt A, Laib A, Ries M, Majumdar S (2003). MicroCT evaluation of normal and osteoarthritic bone structure in human knee specimens. J Orthop Res.

[33] Bobinac D, Spanjol J, Zoricic S, Maric I (2003). Changes in articular cartilage and subchondral bone histomorphometry in osteoarthritic knee joints in humans. Bone..

[34] Reina N, Cavaignac E, Pailhe R, Pailliser A, Bonnevialle N, Swider P (2017). BMI-related microstructural changes in the tibial subchondral trabecular bone of patients with knee osteoarthritis. J Orthop Res.

[35] Akamatsu Y, Koshino T, Saito T, Wada J (1997). Changes in osteosclerosis of the osteoarthritic knee after high tibial osteotomy. Clin Orthop Relat Res.

[36] Takahashi S, Tomihisa K, Saito T (2002). Decrease of osteosclerosis in subchondral bone of medial compartmental osteoarthritic knee seven to nineteen years after high tibial valgus osteotomy. Bull Hosp Jt Dis.

[37] Kroker A, Zhu Y, Manske SL, Barber R, Mohtadi N, Boyd SK (2017). Quantitative in vivo assessment of bone microarchitecture in the human knee using HR-pQCT. Bone..

[38] Starr JF, Bandeira LC, Agarwal S, Shah AM, Nishiyama KK, Hu Y (2018). Robust trabecular microstructure in type 2 diabetes revealed by individual trabecula segmentation analysis of HR-pQCT images. J Bone Miner Res.

[39] Roberts BC, Thewlis D, Solomon LB, Mercer G, Reynolds KJ, Perilli E (2017). Systematic mapping of the subchondral bone 3D microarchitecture in the human tibial plateau: variations with joint alignment. J Orthop Res.

[40] Johnson F, Leitl S, Waugh W (1980). The distribution of load across the knee. A comparison of static and dynamic measurements. J Bone Joint Surg Br Vol.

[41] Harrington IJ (1983). Static and dynamic loading patterns in knee joints with deformities. J Bone Joint Surg Am.

[42] Bhatla JL, Kroker A, Manske SL, Emery CA, Boyd SK (2018). Differences in subchondral bone plate and cartilage thickness between women with anterior cruciate ligament reconstructions and uninjured controls. Osteoarthr Cartil.

[43] Chen Y, Huang YC, Yan CH, Chiu KY, Wei Q, Zhao J (2017). Abnormal subchondral bone remodeling and its association with articular cartilage degradation in knees of type 2 diabetes patients. Bone Res.

[44] Waarsing JH, Bierma-Zeinstra SMA, Weinans H (2015). Distinct subtypes of knee osteoarthritis: data from the osteoarthritis initiative. Rheumatology..

